# A Novel Mittag-Leffler Kernel Based Hybrid Fault Diagnosis Method for Wheeled Robot Driving System

**DOI:** 10.1155/2015/606734

**Published:** 2015-07-02

**Authors:** Xianfeng Yuan, Mumin Song, Fengyu Zhou, Zhumin Chen, Yan Li

**Affiliations:** ^1^School of Control Science and Engineering, Shandong University, Jinan 250061, China; ^2^School of Computer Science and Technology, Shandong University, Jinan 250101, China

## Abstract

The wheeled robots have been successfully applied in many aspects, such as industrial handling vehicles, and wheeled service robots. To improve the safety and reliability of wheeled robots, this paper presents a novel hybrid fault diagnosis framework based on Mittag-Leffler kernel (ML-kernel) support vector machine (SVM) and Dempster-Shafer (D-S) fusion. Using sensor data sampled under different running conditions, the proposed approach initially establishes multiple principal component analysis (PCA) models for fault feature extraction. The fault feature vectors are then applied to train the probabilistic SVM (PSVM) classifiers that arrive at a preliminary fault diagnosis. To improve the accuracy of preliminary results, a novel ML-kernel based PSVM classifier is proposed in this paper, and the positive definiteness of the ML-kernel is proved as well. The basic probability assignments (BPAs) are defined based on the preliminary fault diagnosis results and their confidence values. Eventually, the final fault diagnosis result is archived by the fusion of the BPAs. Experimental results show that the proposed framework not only is capable of detecting and identifying the faults in the robot driving system, but also has better performance in stability and diagnosis accuracy compared with the traditional methods.

## 1. Introduction

In recent years, the wheeled robots have received a wide range of applications and developments [1–3]. Particularly, in home service area, various kinds of wheeled service robots have become members of our family, such as the elderly companion robot [4] and the sweeping robot [5]. However, robot users are usually nonexpert in robot technology, which means that the faults which occurred in the wheeled robot system may cause serious damage to their life and property. The increasing demand of safety, reliability, and the necessity of low cost have become the bottleneck of wheeled robot applications with current technology. Therefore, it is meaningful to focus on novel fault diagnosis methods, particularly for the man-robot coexistent environments.

Generally speaking, the existing fault diagnosis methods can be classified as the model based and the data driven ones [6, 7]. In the earlier days, the research of model based fault diagnosis methods drew much attention and constituted the mainstream of this field [8, 9]. In [10], based on the mathematical model of the robotic manipulator, Caccavale et al. presented a discrete-time framework for diagnosis of sensors and actuators of robotic manipulators. Using particle filter, Yu et al. [11] proposed a fault-proneness prediction method for robot dead reckoning system. Besides the abovementioned methods, the adaptive observer and some other model based methods have also been designed for fault diagnosis of robot platform or robot manipulator [12, 13]. Those model based fault diagnosis methods are effective and suitable for the diagnosis problem of robot manipulator or robot arm, because robot arm usually works in a structured environment and it is relatively easier to get the accurate mathematical model. While, for wheeled robots, firstly, their working environments are usually dynamic and unstructured, secondly, wheeled robots are usually equipped with various kinds of equipment that are more complex in both hardware and software aspects compared with manipulators. Thus it is hard to get an accurate mathematical model of a wheeled robot working in an unstructured environment, which becomes a restriction of those model based methods. Moreover, the wheeled robots are well equipped with multiple sensors which implies that large data volumes containing robot running status information are available. Those large data volumes imply difficulties in system modeling, while they provide the required information for data driven based fault diagnosis method.

Principal component analysis (PCA) is a typical representative of the data driven fault diagnosis method. PCA is more suitable for fault detection rather than diagnosis, because it does not use the input-output relationships [14]. Therefore, in order to improve the diagnosis ability, PCA is often used by combining the classifiers, such as the neural network (NN) and the support vector machine (SVM). This hybrid method has been applied in the fault diagnosis of rotating machinery [15], power transmission systems [16], and some other aspects [17, 18]. Applications of PCA could be useful in extracting and interpreting process information from massive data sets, and the pattern recognition techniques could also be used to diagnose the specific running status of the robot.

Nevertheless, there are mainly two problems that exist in the above hybrid diagnosis methods. On the one hand, most of the studies adopted the existing classical kernel (e.g., Gaussian kernel and polynomial kernel) as the kernel function of SVM in their diagnosis methods, while new kernel functions with better classification performance need to be proposed, proved, and applied to the robot fault diagnosis fields. On the other hand, the diagnosed objects are usually complex and with varying degrees of uncertainties. A single PCA model cannot achieve full and complete awareness of the diagnosed object so that the information fusion in data level or decision level is needed to reduce the existing uncertainties.

Mittag-Leffer functions [19, 20] play fundamental roles in fractional calculus, which exhibit intermediate process among exponential function, power function, and polynomial function. Nowadays, fractional calculus has been successfully applied in many aspects, such as the application of fractional Fourier transform in signal processing [21] and the application of fractional order PI controllers [22]. Inspired by fractional calculus, a novel fractional Gaussian kernel named ML-kernel is proposed in this paper, which is a generalization of the traditional Gaussian kernel. The proposed ML-kernel is proved to be positive definite and its diagnosis performance is discussed in this paper. Besides, a hybrid fault diagnosis framework is discussed for robot driving system based on Dempster-Shafer (D-S) fusion and ML-kernel support vector machine (SVM). Multiple PCA models are established to do fault feature extraction and the fault feature vectors are used as the inputs of the ML-kernel SVM classifiers. The ML-kernel SVM classifiers output the preliminary fault diagnosis results which are fused by D-S fusion and the fusion result is taken as the final diagnosis result. Two sets of comparative experiments are carried out to validate the proposed method.

The remainder of this paper is organized as follows.  Section 2 briefly introduces the SVM method and the positive definiteness of the presented ML-kernel is also proved in this section. In Section 3, the proposed fault diagnosis framework is described in detail.  Section 4 illustrates the architecture of the experimental wheeled robot driving system and the application studies for various fault conditions.  Section 5 is devoted to conclusions.

## 2. SVM Algorithm and the Presented ML-Kernel Function

### 2.1. Conventional SVM Algorithm

In the past few years, SVM has been one of the most highly studied topics in the machine learning fields, and it has been successfully applied in practice, especially for classification problems (e.g., fault diagnosis) [23, 24]. Based on the statistical theory of VC dimension and structural risk minimization inductive principle, SVM reaches the best compromise between the complexity of modeling and the leaning ability and hunts the best generalization ability. The basic SVM [25] deals with linearly separable two class cases and it can cope with nonlinear problems by introducing kernel functions and slack penalty. Given a training set *S*_*t*_ = {*x*_*i*_, *y*_*i*_}_*i*=1_^*N*^, where *x*_*i*_ ∈ *R* ^*d*^ is the *i*th training input vector, *N* is the number of training data for SVM, *d* is the dimension of the input data, and *y*_*i*_ ∈ {−1,1} is the set of classification tag for training. The optimal hyperplane separating the data can be obtained as a solution to the following optimization problem:

(1) min⁡ω,b,ξi 12ωTω+c∑i=1Nξi,s.t. yiωTxi+b≥1−ξi,ξi≥0,  i=1,2,…,N,

where *c* is the slack penalty, *ω* is the adjustable weight vector, *b* is the offset of the hyperplane, and *ξ*_*i*_ is the distance between the margin and the *x*_*i*_ lying on the wrong side. The equivalent Lagrangian dual problem can be described as follows:

(2) min⁡αi ∑i=1Nαi−12∑i,j=1NyiyjαiαjxiTxj,s.t. ∑i=1Nαiyi=0,0≤αi≤C,  i=1,2,…,N,

where *α*_*i*_ is the Lagrangian coefficient, from which we can obtain *ω* = ∑_*i*=1_^*N*^ *α*_*i*_ *y*_*i*_ *x*_*i*_, *b* = *y*_*i*_ − *ω* ^*T*^ *x*_*i*_, to solve (1).

The kernel function can map the input vector *x* into feature space and returns a dot product of the feature space. The linear discriminant function with kernel *K*(*x*_*i*_, *x*_*j*_) is given by the following:

(3) $$\begin{array}{c} f\left(\;x\;\right)=\mathrm{sgn}\left(\;\overset{N}{\underset{i,j=1}{\sum }}a_{i}y_{i}K\left(\;x_{i},x_{j}\;\right)+b\;\right), \end{array}$$

where sgn⁡(·) is the signum function.

The fault diagnosis of a robot driving system is a multiple class classification problem, while the conventional SVM was designed for the binary classification problem, so it is not suitable for the fault diagnosis in its original form. A few types of methods for multiclass SVM have been proposed [26]: one against one, one against others, direct acyclic graph, and so forth. This study employs the “one against one” multiclass SVM. In order to construct the BPAs, we need the probabilistic outputs of the SVM classifiers and the “pairwise coupling” method [27] is used to solve this problem.

### 2.2. Kernel Function

The nonlinear pattern recognition problem in fault diagnosis can be transformed into the linear problem in some very high-dimensional feature spaces. The kernel function *K*(*x*_*i*_, *x*_*j*_) = *ϕ*(*x*_*i*_)^*T*^ *ϕ*(*x*_*j*_) is able to handle any dimension feature spaces without the accurate calculation of *ϕ*(*x*_*i*_) and *ϕ*(*x*_*j*_). It has been proven that any function that satisfies the Mercer theorem can be used as a kernel function [28]. Currently, there are three typical kinds of kernel functions:

(1) Polynomial kernel function

(4) $$\begin{array}{c} K\left(\;x_{i},x_{j}\;\right)={\left[\;\left(\;x_{i},x_{j}\;\right)+1\;\right]}^{d}. \end{array}$$

(2) Radial basis kernel function (RBF)

(5) $$\begin{array}{c} K\left(\;x_{i},x_{j}\;\right)=\exp\left(\;-\frac{{\left\|x_{i}-x_{j}\right\|}^{2}}{2\delta^{2}}\;\right). \end{array}$$

(3) Sigmoid kernel function

(6) $$\begin{array}{c} K\left(\;x_{i},x_{j}\;\right)=\tanh\left(\;x_{i}^{T}x_{j}+b\;\right). \end{array}$$

### 2.3. Proof of the Positive Definiteness of the ML-Kernel

As the core of SVM, kernel function and the parameters of the model determine the performance of the SVM algorithm applied to the fault diagnosis system. In this paper, we employ the Mittag-Leffler function as a novel kernel function named as ML-kernel. The Mittag-Leffler function [29] is defined as follows:

(7) $$\begin{array}{c} E_{\alpha }\left(\;z\;\right)=\overset{\infty }{\underset{k=0}{\sum }}\frac{z^{k}}{\Gamma \left(k\alpha +1\right)}, \end{array}$$

where Γ is the Gamma function and *α* is an arbitrary positive constant. For *α* = 1, (7) becomes *E*_1_(*z*) = *e* ^*z*^. The presented ML-kernel function can be defined as

(8) $$\begin{array}{c} K\left(\;x_{i},x_{j}\;\right)=E_{\alpha }\left(\;-\frac{{\left\|x_{i}-x_{j}\right\|}^{2\alpha }}{2\delta^{2\alpha }}\;\right), \end{array}$$

where 0 < *α* ≤ 1. When *α* = 1, (8) becomes *E*_1_(−‖*x*_*i*_ − *x*_*j*_‖^2^/2*δ* ^2^) = exp(−‖*x*_*i*_ − *x*_*j*_‖^2^/2*δ* ^2^), and the ML-kernel is identical to the Gaussian RBF kernel in this condition.

Given a kernel, it is in general straightforward to verify its continuity and symmetry, while the positive definiteness is more important and essential for a kernel. Thus, the proof of the positive definiteness of the proposed ML-kernel is given as below.

For convenience, letting *t* = ‖*x*_*i*_ − *x*_*j*_‖^2^/2*δ* ^2^, the ML-kernel (8) can be written as *K*(*x*_*i*_, *x*_*j*_) = *E*_*α*_(−*t* ^*α*^). In Laplace domain, we can get [30]

(9) Kxi,xjEα−tα=L−1sα−1sα+1=12πilimT→∞⁡∫γ−iTγ+iTestsα−1sα+1ds,

where *γ* is a real number that keeps the contour path of integration which is in the region of convergence of *s* ^*α*−1^(*s* ^*α*^ + 1), *i* ^2^ = −1, and *ℒ* ^−1^(·) denotes the inverse Laplace transformation.

The integration path in (9) can be bended into the equivalent Hankel contour *H*_*a*_(*δ*), which contains three parts: one line *L*_1_ that starts from (−*∞*, −*δ*), an arc *C* that encircles the circular disc |*s* | = *δ* ≤ *γ* counterclockwise, and the other line *L*_2_ that ends at (−*∞*, *δ*). So, (9) can be written as

(10) Eα−tα12πi∫Haδestsα−1sα+1ds=I1+I2+I3=12πi∫L1estsα−1sα+1ds+12πi∫Cestsα−1sα+1ds+12πi∫L2estsα−1sα+1ds.

Along *L*_1_, we have *s* = *re* ^−*πi*^ = −*r*, *δ* ≤ *r* ≤ *∞*, and as *s* goes from −*∞* to −*δ*, *r* goes from +*∞* to *δ*,

(11) $$\begin{array}{c} I_{1}=\frac{1}{2\pi i}\overset{-\delta }{\underset{-\infty }{\int }}\frac{e^{st}s^{\alpha -1}}{s^{\alpha }+1}ds=-\frac{1}{2\pi i}\overset{+\infty }{\underset{\delta }{\int }}\frac{e^{-rt}r^{\alpha -1}e^{-\alpha \pi i}}{r^{\alpha }e^{-\alpha \pi i}+1}dr. \end{array}$$

Along *L*_2_, we have *s* = *re* ^*πi*^ = −*r*, *δ* ≤ *r* ≤ *∞*, and as *s* goes from −*δ* to −*∞*, *r* goes from *δ* to +*∞*:

(12) $$\begin{array}{c} I_{3}=\frac{1}{2\pi i}\overset{-\infty }{\underset{-\delta }{\int }}\frac{e^{st}s^{\alpha -1}}{s^{\alpha }+1}ds=\frac{1}{2\pi i}\overset{+\infty }{\underset{\delta }{\int }}\frac{e^{-rt}r^{\alpha -1}e^{\alpha \pi i}}{r^{\alpha }e^{\alpha \pi i}+1}dr. \end{array}$$

Along *C*, we have *s* = *δe* ^*iθ*^, *θ* ∈ (−*π*, *π*), and

(13) I212πi∫Cestsα−1sα+1ds=12πi∫−ππereiθtreiθα−1reiθα+1rieiθdθ=0.

Hence, we can obtain

(14) I1+I3=12πi∫δ+∞−rα−1e−απirαe−απi+1e−rt+rα−1eαπirαeαπi+1e−rtdr=12πi∫δ+∞rα−1rα+e−απi−rα−1rα+eαπie−rtdr=12πi∫δ+∞rα−1eαπi−e−απir2α+rαeαπi+e−απi+1e−rtdr=1π∫δ+∞rα−1sin⁡απr2α+2rαcos⁡απ+1e−rtdr.

Letting *δ* → 0^+^, we have

(15) Eα−tαI1+I2+I3=1π∫δ+∞rα−1sin⁡απr2α+2rαcos⁡απ+1e−rtdr>0.

Therefore *K*(*x*_*i*_, *x*_*j*_) = *E*_*α*_(−‖*x*_*i*_ − *x*_*j*_‖^2*α*^/2*δ* ^2*α*^) = *K*(*x*_*j*_, *x*_*i*_), and, for all *a* ∈ *ℝ*, we have ∑_*i*,*j*=1_^*n*^ *a*_*i*_ *a*_*j*_ *K*(*x*_*i*_, *x*_*j*_) ≥ 0. In other words, the proposed ML-kernel is symmetrical and positive definite. Therefore, the proof is complete.

## 3. Fault Diagnosis Method Based on ML-Kernel SVM and D-S Fusion

**Figure 1:**
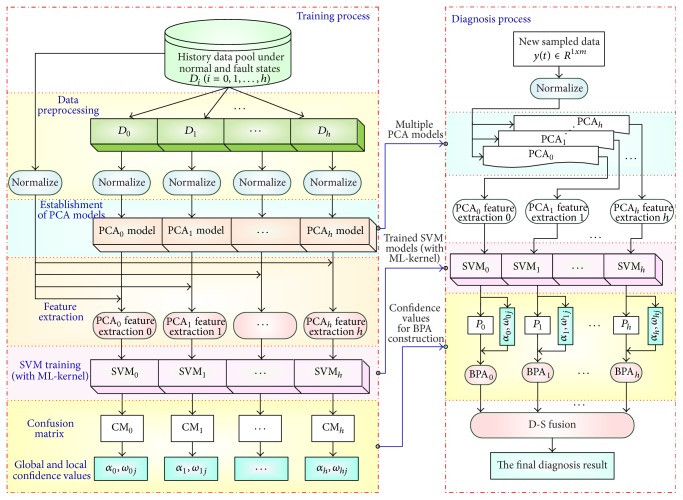
Flow diagram of the proposed fault diagnosis approach.

As shown in Figure 1, there are two main processes of the proposed approach, namely, the training process and the fault diagnosis process. Before the application of the proposed approach, the initial samples should be obtained from the laboratory experiments. In the training process, multiple PCA models are set up based on the data sampled in the normal and faulty states. Then, those models are used to do fault feature extraction and the ML-kernel SVM classifiers are trained. In the diagnosis process, new sampled data are normalized firstly. Secondly, the PCA models established in the training process are applied to do fault feature extraction. The fault feature vectors are then served as the inputs of the trained ML-kernel SVM classifiers, respectively, and the probabilistic outputs of the classifiers are taken as the preliminary fault diagnosis results. The BPAs are constructed based on the preliminary fault diagnosis results and the confidence values calculated from the confusion matrix. To reduce the uncertainties of the preliminary diagnosis results, the D-S fusion algorithm is introduced for decision fusion and the final diagnosis results are given based on the fusion results. The proposed approach is elaborated in detail as follows.

### 3.1. Data Preprocessing and Establishment of Multiple PCA Models

Suppose that there are *h* + 1 kinds of robot running states represented as {*S*_0_, *S*_1_,…, *S*_*h*_}. *S*_0_ represents the normal condition and *S*_1_,…, *S*_*h*_ represent *h* kinds of faulty states. Given that the robot driving system is equipped with *m* sensors and we get *n* groups of samples in each running state, so the original sampled data sets can be written as *D*_all_ = [*D*_0_,…, *D*_*k*_,…, *D*_*h*_]^*T*^, *D*_*k*_ ∈ *R* ^*n*×*m*^  (*k* = 0,1,…, *h*). *D*_0_ represents the samples under normal condition *S*_0_ and *D*_*k*_  (*k* = 1,…, *h*) represent the samples in the *k*th kind of faulty state *S*_*k*_. To establish PCA models, several steps are introduced.

**Step 1 (data normalization).**

To reduce the influence of different dimensions of the sensors, the training data should be normalized before establishing the PCA models. For a data set of *n* observations and *m* process variables *D*_*k*_ ∈ *R* ^*n*×*m*^(*k* = 0,1,…, *h*), we can get the normalized data matrix ${\bar{D}}_{k}$ by

(16) Mj=1n∑i=1ndij,σj=1n−1∑i=1ndij−Mj2,x¯ij=dij−Mjσj,

where *M*_*j*_ and *σ*_*j*_ are the mean and standard deviation, respectively, of the *j*th variable, *d*_*ij*_ is an element of matrix *D*_*k*_, and ${\bar{x}}_{ij}$ is an element of the normalized data matrix ${\bar{D}}_{k}$.

**Step 2 (singular value decomposition).**

Consider

(17) $$\begin{array}{c} C_{k}=P_{k}\Lambda_{k}P_{k}^{T}, \end{array}$$

where *C*_*k*_ represents covariance matrix of ${\bar{D}}_{k}$, Λ_*k*_ is a diagonal matrix containing the eigenvalues of *C*_*k*_ in decreasing order, and *P*_*k*_ is orthogonal and contains the eigenvectors of *C*_*k*_.

**Step 3 (determine the loading matrix according to the number of PCs).**

Given *β*_*i*_ = *λ*_*i*_/∑_*j*=1_^*m*^ *λ*_*j*_, the number of principal components (PCs) *l* is determined to satisfy the equation *β*_1_ + *β*_2_ + ⋯+*β*_*l*_ ≥ *μ*, where *μ* is a constant and usually required to be bigger than 0.85 [31].

The loading matrix ${\hat{P}}_{k}=[p_{1},p_{2},\ldots ,p_{l}]$ consists of the former *l* eigenvectors of the covariance matrix and ${\bar{D}}_{k}$ can be decomposed as

(18) $$\begin{array}{c} {\bar{D}}_{k}=T_{s}{\hat{P}}_{k}^{T}+E, \end{array}$$

where $T_{s}={\bar{D}}_{k}{\hat{P}}_{k}$ is named as score matrix and *E* is the residual matrix.

### 3.2. Feature Extraction and SVM Training

During the process of PCA, the orthogonal loading matrix ${\hat{P}}_{k}$ can be considered as the main features of the original training data set. So, we can do data dimensionality reduction and feature extraction at the same time using the following equation:

(19) $$\begin{array}{c} F_{k}=\left(\;{\bar{D}}_{\text{all}}\cdot {\hat{P}}_{k}\;\right)\in R^{\left(h+1\right)n\times l},\;k=0,1,\ldots ,h, \end{array}$$

where ${\bar{D}}_{\text{all}}\in R^{(h+1)n\times m}$ is the normalized training data sets and ${\hat{P}}_{k}\in R^{m\times l}$ is the loading matrix of the *k*th PCA model. *l* is the number of principal components and *F*_*k*_ is used as the final training sets of SVM_*k*_  (*k* = 0,1,…, *h*).

A novel ML-kernel presented in Section 2.3 is applied as the kernel function of SVM_*k*_ and particle swarm optimization (PSO) [32] is adopted to tune the parameters *c*, *δ*, and *α*. Here, *c* is the slack penalty and *δ* and *α* are two parameters of the ML-kernel.

### 3.3. Decision Fusion

To reduce the uncertainties and imprecisions of the preliminary fault diagnosis results, D-S fusion is introduced in the proposed fault diagnosis framework. The determination of BPAs is the first and most important step in evidence theory. In our approach, we construct BPAs based on the probabilistic outputs of the PSVM classifiers and their confidence values.

**Step 1 (calculation of the confidence values).**

The average classification accuracy of SVM_*k*_ can be calculated by

(20) $$\begin{array}{c} {\bar{a}}_{k}=\frac{\sum_{p=1}^{h+1}\left(c_{pp}\cdot N_{p}\right)}{N}, \end{array}$$

where *c*_*pp*_ is the diagonal elements of the confusion matrix of SVM_*k*_, *N*_*p*_ = *n* is the number of samples under the *p*th kind of fault condition, and *N* = (*h* + 1)*n* is the total number of training samples in the training set *F*_*k*_. So, we can get ${\bar{a}}_{k}=\sum_{p=1}^{h+1}c_{pp}/(h+1)$, which can be used as the global confidence of SVM_*k*_.

The *q*th column vector of the confusion matrix *c*_·*q*_  (*q* = 1,2,…, *h* + 1) indicates the local confidence for the *q*th kind of fault and the local confidence can be calculated by

(21) $$\begin{array}{c} \omega_{kq}=\frac{c_{qq}}{\sum_{p=1}^{h+1}c_{pq}},\;q=1,2,\ldots ,h+1. \end{array}$$

Then we can incorporate the local confidence *ω*_*kq*_ into the probabilistic output of SVM_*k*_ and after normalization we can get

(22) $$\begin{array}{c} f_{kq}^{\prime }=\frac{\omega_{kq}f_{kq}}{\sum_{q=1}^{h+1}\omega_{kq}f_{kq}}, \end{array}$$

where 0 ≤ *f*_*kq*_ ≤ 1 is the output of SVM_*k*_.

**Step 2 (construction of BPAs and D-S fusion).**

In our approach, the BPAs are defined as

(23) mk⌀,S0,S1,…,Sh,Θ=0,a¯kfk0′,a¯kfk1′,…,a¯kfkh′,1−a¯k.

It can be indicated from (23) that the frame of discernment *P*(Θ) = {*⌀*, *S*_0_, *S*_1_,…, *S*_*h*_, Θ}. Here, *⌀* denotes the empty set, and *S*_*h*_ represents the *h*th kind of running condition of the robot. It is obvious that ∑_*A*∈*P*(Θ)_ *m*_*k*_(*A*) = 1; *m*_*k*_(*⌀*) = 0. With BPAs, we use a fast fusion algorithm based on the matrix analysis [33] to accomplish D-S fusion algorithm.

## 4. Implementations on Wheeled Robot

A real application of robot driving system fault diagnosis is selected to illustrate the aforesaid theories and the proposed diagnosis framework. The experimental robot and its fault diagnosis problem are described briefly, followed by the discussions of the three key components in the proposed diagnosis framework, namely, data collection and preprocessing, feature extraction and SVM training, and decision fusion. In addition, several groups of contrast experiments are given in this section.

### 4.1. Description of Experimental Robot

**Figure 2:**
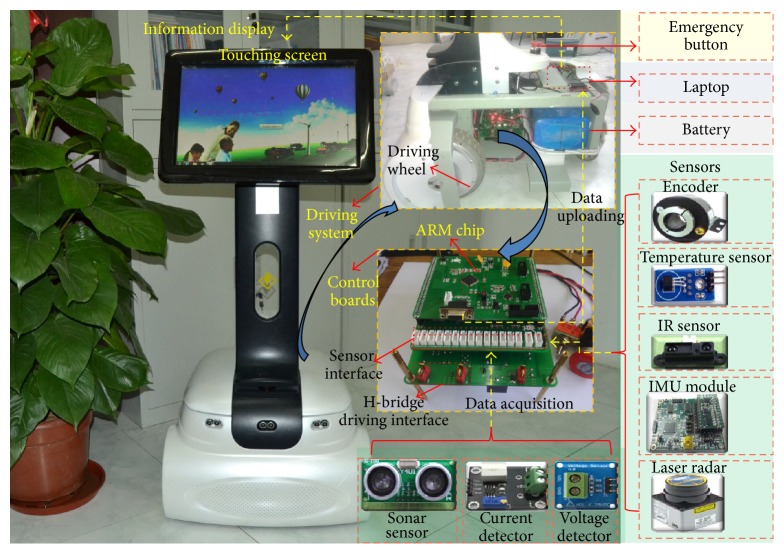
Experimental robot.

**Figure 3:**
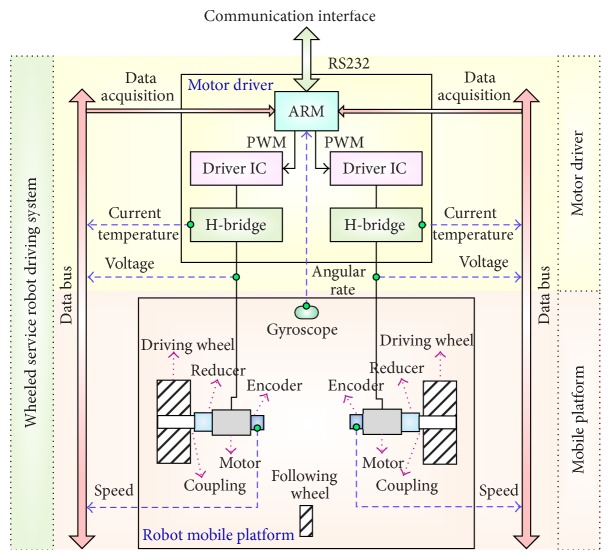
Architecture of wheeled robot driving system.

As shown in Figure 2, we use the wheeled service robot developed by our research group as the experimental platform. This robot is driven by two differential wheels and it is equipped with various kinds of sensors such as one gyroscope (L3GD20), two incremental encoders, two temperature sensors (DS18B20), current detecting circuits, and voltage detecting circuits. The architecture of the driving system is shown in Figure 3.

In general, faults which occurred in a wheeled robot driving system can be divided into two categories: mechanical faults and sensor faults. In fact, each of the two categories can be subdivided into many small classes. However, only a few typical kinds of high risk faults often occur in the actual course of using the robot [11]. In this paper, we mainly focus on the diagnosis of 7 common kinds of high risk faults and the normal condition *S*_0_ is treated as a special kind of “fault.” As shown in Table 1, the fault space can be defined as *S*_err⁡_ = {*S*_0_, *S*_1_,…, *S*_7_}.

In order to achieve the effective detection and diagnosis of the faults presented in Table 1, the fault symptom space must be determined, which means that we should select the available and useful sensor signals in the robot driving system. It can be indicated from Figure 3 that the working status of the robot driving system is closely related with motor speed, driving voltage, armature current, angular rate, and temperature of H-bridge. Considering that the temperature of H-bridge is easily affected by environment temperature, we use the change rate of the H-bridge temperature as characteristic signal and the final fault symptom space can be defined as {left wheel speed **V**_*l*_, right wheel speed **V**_*r*_, left motor driving voltage **U**_*l*_, right motor driving voltage **U**_*r*_, left motor armature current **I**_*l*_, right motor armature current **I**_*r*_, change rate of the left H-bridge temperature **T**_*l*_, change rate of the right H-bridge temperature **T**_*r*_, angular rate **W**}.

### 4.2. Data Collection and Preprocessing

**Figure 4:**
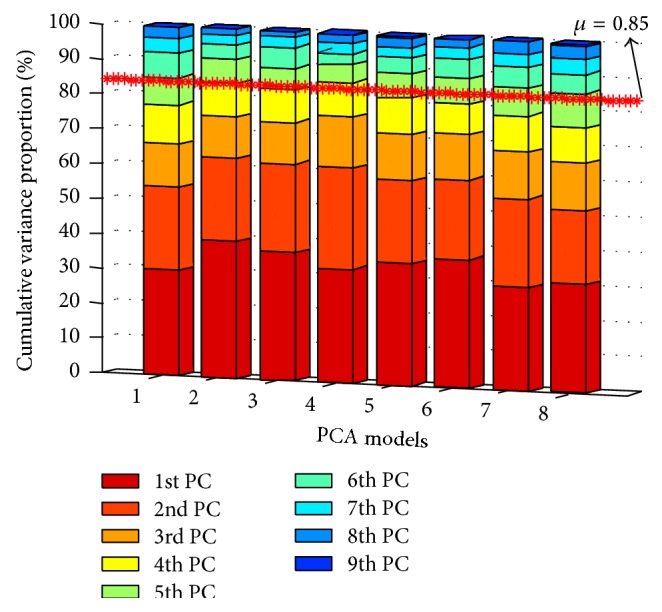
Cumulation variance proportion of the PCs.

The robot motion controller (ARM chip) is responsible for data collection and uploading. In our experiments, we sample 200 sets of data under each of the running conditions (*S*_0_–*S*_7_), respectively. So, the raw data sets can be marked as *D*_all_ = [*D*_0_,…, *D*_*k*_,…, *D*_7_]^*T*^ ∈ *R* ^1600×9^ and *D*_*k*_ ∈ *R* ^200×9^ denotes data sampled under the *k*th running condition. For simplification without losing generality, 100 sets of data in each *D*_*k*_ are randomly selected as the original training samples *X*_*k*_ ∈ *R* ^100×9^ and the remaining 100 sets of data are used as the original testing samples *Y*_*k*_ ∈ *R* ^100×9^. With the normalized ${\bar{X}}_{k}\;\;(k=0,\ldots ,7)$, 8 PCA models are established by (17) and (18). The cumulation variance proportion of the PCs for each PCA_*k*_ model is shown in Figure 4 and the threshold value *μ* is set to 0.85. We can get *l*_*k*_ = [5,5, 5,4, 5,5, 5,5], which represents the optimal number of PCs for PCA_*k*_  (*k* = 0,…, 7).

### 4.3. Feature Extraction and SVM Training

The normalized training data set ${\bar{X}}_{\text{all}}={\left[{\bar{X}}_{0},\ldots ,{\bar{X}}_{7}\right]}^{T}\in R^{800\times 9}$ is projected onto the principal component subspace of each PCA_*k*_ model and we can get the feature vectors *F*_*k*_ ∈ *R* ^800×*l*_*k*_^, (*k* = 0,…, 7) by (19). Then *F*_*k*_ is used to train SVM_*k*_ with 5-fold cross validation and PSO algorithm for parameters optimization.

**Figure 5:**
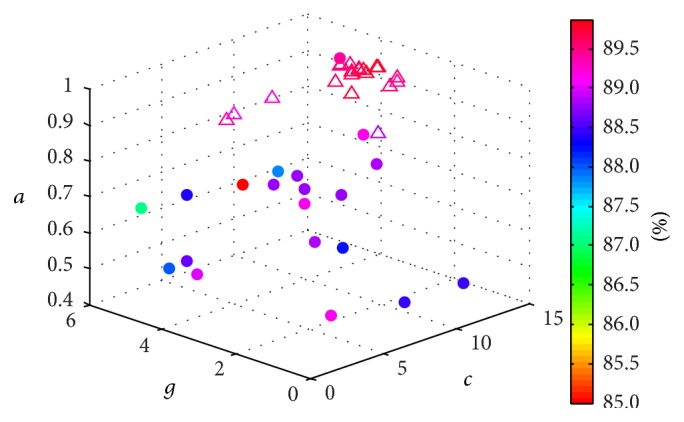
Parameters tuning based on PSO algorithm (∙ denotes the original distribution of the particles and △ denotes the final distribution of the particles).

**Figure 6:**
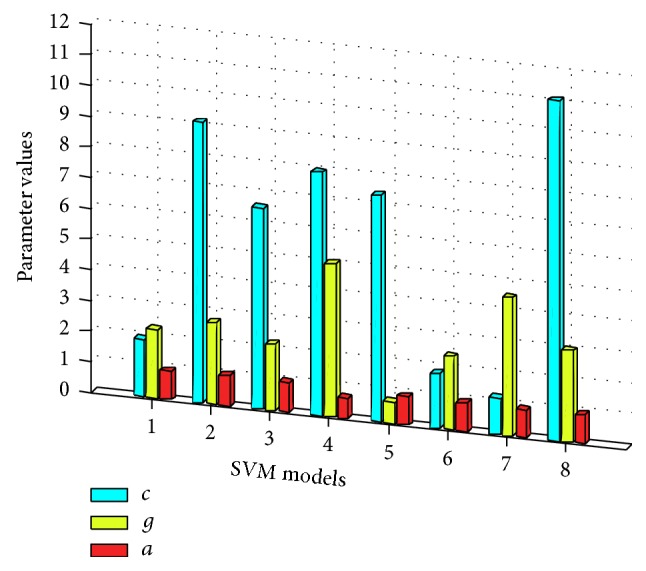
Optimal parameters of SVM_*k*_.

**Figure 7:**
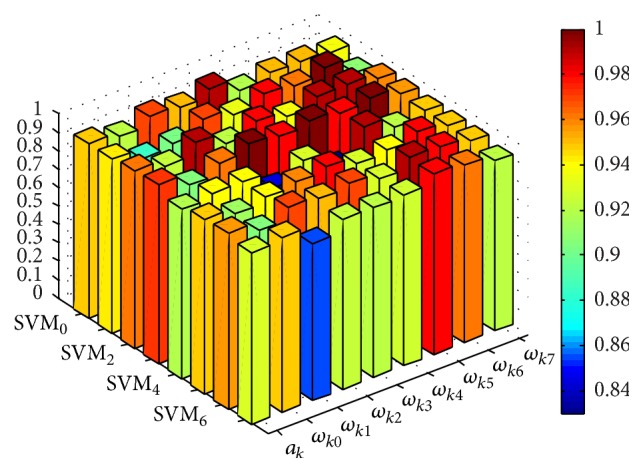
Confidence values of SVM_*k*_.

Taking SVM_8_ as an instance, Figure 5 shows the distribution of the particles during parameters optimization process using PSO algorithm and the optimal parameters of SVM_8_ are {*c* = 11.086, *g* = 2.997, *α* = 0.950}. The optimal parameters of other SVM models are shown in Figure 6. With the trained SVM_*k*_ models, the global and local confidence values (${\bar{a}}_{k},\omega_{k0},\ldots ,\omega_{k7}$) can be obtained using (20) and (21), respectively, and the confidence values of SVM_*k*_  (*k* = 0,…, 7) are presented in Figure 7.

### 4.4. Decision Fusion

With the global and local confidence values elaborated in Figure 7, we can construct the BPAs for D-S fusion by (23). Taking *S*_2_ and *S*_4_, for example, we get two sets of fusion records randomly and the details are presented in Table 2.

As shown in Table 2, there are three error diagnoses in the 7th, the 14th, and the 16th row, because one single PCA model cannot achieve complete awareness of the robot driving system. While in the proposed approach, multiple PCA models are used to do feature extraction and D-S fusion is applied to fuse the outputs of the ML-kernel PSVM classifiers. Thus, the proposed approach can achieve better awareness of the system and diagnose the faults accurately. Besides, it can be indicated from the 5th and the 7th column that the confidence value (0.959 and 0.995) after D-S fusion is much bigger than that of any single PCA_*k*_. In other words, the proposed approach can reduce the uncertainties of the diagnosis result efficiently.

The classification accuracy indicates the ability to diagnose the entire categories which are defined as 7 kinds of faulty states {*S*_1_,…, *S*_7_} and 1 normal state {*S*_0_}. In this study, we use the true positive rate as the diagnosis accuracy, which is defined as

(24) $$\begin{array}{c} \text{Acc}=\frac{N_{\text{TP}}}{N_{\text{TP}}+N_{\text{FN}}}\times 100\%, \end{array}$$

where *N*_FN_ is the number of false negatives defined as the number of faults in category *k* that are not classified as category *k* and *N*_TP_ is the number of true positives.

**Figure 8:**
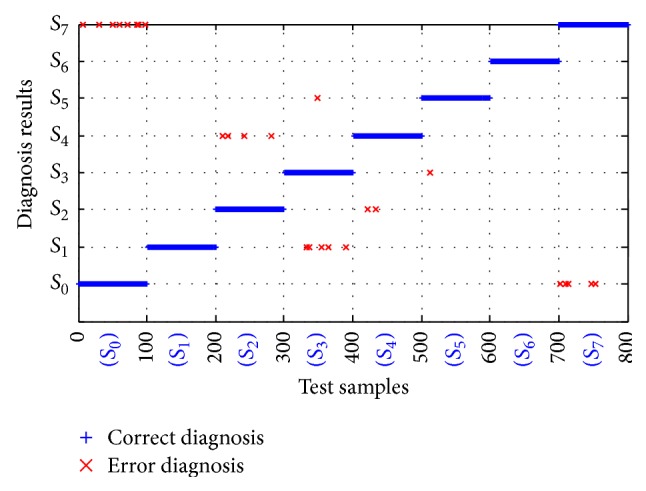
Fault diagnosis result.

According to Figure 8, the final diagnosis accuracy is 96.75% for the testing samples *Y*_all_ = [*Y*_0_,…, *Y*_*k*_,…, *Y*_7_] ∈ *R* ^800×9^ (see Section 4.2) in our experiments.

### 4.5. Contrast Experiments

To further verify the effectiveness of the proposed framework and the ML-kernel, several groups of contrast experiments are conducted, respectively, for comparison.

#### 4.5.1. Evaluation of the ML-Kernel

In order to evaluate the performance of the proposed ML-kernel, we compare the diagnosis accuracy of the ML-kernel with the existing three typical kernel functions based on the proposed hybrid diagnosis framework. For fair comparison, we use the same sets of training samples *X*_all_ = [*X*_0_,…, *X*_7_] and testing samples *Y*_all_ = [*Y*_0_,…, *Y*_7_] (mentioned in Section 4.2). Besides, PSO and 5-fold cross validation are applied to find the optimal parameters for each kernel function. The experimental results are presented in Table 3.

From the 5th row and the 6th row of Table 3, we can see that the proposed ML-kernel has an identical classification performance compared with the classical Gaussian RBF kernel when *α* = 1. When 0 < *α* ≤ 1, we can see that the diagnosis ability of the proposed ML-kernel is better than that of the classical Gaussian RBF kernel. From the discussion in Section 2.3, we know that the proposed ML-kernel can be regarded as a generalized form of the Gaussian RBF kernel and the experimental results here verify this conclusion again. Table 3 demonstrates that the proposed ML-kernel has the best performance for fault diagnosis of the wheeled robot driving system, followed by the Gaussian RBF kernel and the polynomial kernel, while the sigmoid kernel has the worst performance in our experiments.

#### 4.5.2. Evaluation of the Proposed Hybrid Diagnosis Framework

**Figure 9:**
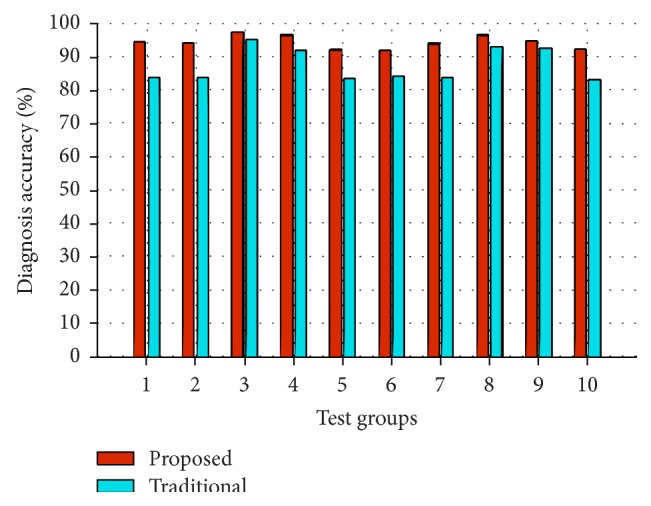
Experimental results based on different frameworks.

The performance of the proposed framework can be evaluated by comparison with traditional nonfusion diagnosis framework. 10 groups of new test data are sampled and each group contains 800 samples which are sampled under each of the running conditions (*S*_0_,…, *S*_7_), respectively (100 samples for each running condition). The ML-kernel is adopted as the kernel function of the SVMs both in the proposed framework and in the traditional framework. The experimental result is shown in Figure 9, from which we can see that the proposed framework achieves the average accuracy of 94.46% (where the highest diagnosis accuracy and the standard deviation are 97.5% and 1.95, resp.). While, for the traditional framework, the average accuracy is 88.15% (where the highest diagnosis accuracy and the standard deviation are 95.5% and 5.76, resp.), it is clear that the proposed framework achieves better performance both in diagnosis accuracy and in stability, which can be owing to the multiple PCA models and the fusion in decision level.

## 5. Conclusion

A novel hybrid fault diagnosis framework for wheeled robot driving system is proposed in this paper. The proposed framework is composed of three key components, namely, data collection and preprocessing, feature extraction and SVM training, and decision fusion. Besides, a novel fractional ML-kernel is presented and its positive definiteness and diagnosis ability are discussed in this study. In the proposed framework, multiple PCA models are established to do fault feature extraction firstly. Secondly, the extracted fault feature vectors are used to train the ML-kernel PSVM classifiers with PSO algorithm and cross validation for parameters tuning. Based on the probabilistic outputs and confidence values of those classifiers, the BPAs are constructed. Finally, the BPAs are fused by D-S fusion algorithm that follows the final diagnosis result. In contrast with the earlier studies, the proposed approach can achieve better awareness of the diagnosed system and reduce the uncertainties of the diagnosis result significantly. Through an illustrative application of wheeled robot driving system fault diagnosis, the proposed method is verified as an efficient way of diagnosing the faults in robot driving system and has better performance in stability (standard deviation 1.95) and diagnosis accuracy (highest diagnosis accuracy 97.5%) compared with the traditional methods. In the future, the combination with parallel computing and the cost-sensitive fault diagnosis framework will be studied.

## Figures and Tables

**Table 1:** Fault position and its common fault modes.

Fault categories	Fault position	Fault mode	Tag
Normal condition	None	None	*S*_0_

Mechanical faults	Left wheel	Low pressure	*S*_1_
	Right wheel	Low pressure	*S*_2_
	Left coupling	Loosening	*S*_3_
	Right coupling	Loosening	*S*_4_

Sensor faults	Left encoder	Pulse loss	*S*_5_
	Right encoder	Pulse loss	*S*_6_
	Gyroscope	Constant drift	*S*_7_

**Table 2:** BPAs assignments and fusion experiment records.

Fault mode	PCA model	BPAs									Outputs
		m(*S*_0_)	m(*S*_1_)	m(*S*_2_)	m(*S*_3_)	m(*S*_4_)	m(*S*_5_)	m(*S*_6_)	m(*S*_7_)	m(θ)	
*S*_2_	PCA_0_	0.004	0.003	**0.463**	0.003	0.446	0.002	0.024	0.004	0.051	*S*_2_
	PCA_1_	0.004	0.004	**0.492**	0.006	0.333	0.003	0.095	0.005	0.058	*S*_2_
	PCA_2_	0.004	0.003	**0.620**	0.004	0.279	0.002	0.046	0.003	0.039	*S*_2_
	PCA_3_	0.001	0.001	**0.708**	0.001	0.151	0.001	0.107	0.002	0.028	*S*_2_
	PCA_4_	0.004	0.002	**0.350**	0.002	*0.546 *	0.002	0.010	0.003	0.081	*S*_4_
	PCA_5_	0.004	0.002	**0.498**	0.004	0.410	0.002	0.025	0.004	0.051	*S*_2_
	PCA_6_	0.002	0.002	**0.507**	0.002	0.390	0.002	0.047	0.003	0.045	*S*_2_
	PCA_7_	0.003	0.002	**0.609**	0.003	0.301	0.002	0.006	0.003	0.071	*S*_2_
	DS	0.000	0.000	**0.959**	0.000	0.041	0.000	0.000	0.000	0.000	*S*_2_

*S*_4_	PCA_0_	0.007	0.004	0.419	0.006	**0.501**	0.002	0.005	0.005	0.051	*S*_4_
	PCA_1_	0.005	0.003	0.021	0.004	**0.902**	0.002	0.001	0.004	0.058	*S*_4_
	PCA_2_	0.006	0.003	*0.515 *	0.005	**0.423**	0.002	0.003	0.004	0.039	*S*_2_
	PCA_3_	0.001	0.001	0.022	0.001	**0.942**	0.000	0.003	0.002	0.028	*S*_4_
	PCA_4_	0.007	0.003	*0.742 *	0.004	**0.153**	0.002	0.002	0.006	0.081	*S*_2_
	PCA_5_	0.007	0.003	0.320	0.005	**0.605**	0.002	0.002	0.005	0.051	*S*_4_
	PCA_6_	0.004	0.003	0.400	0.003	**0.535**	0.002	0.004	0.004	0.045	*S*_4_
	PCA_7_	0.005	0.003	0.363	0.005	**0.544**	0.002	0.002	0.005	0.071	*S*_4_
	DS	0.000	0.000	0.005	0.000	**0.995**	0.000	0.000	0.000	0.000	*S*_4_

**Table 3:** Diagnosis accuracy of the proposed hybrid framework with different kernel functions.

Kernel function	Diagnosis accuracy								
	*S*_0_	*S*_1_	*S*_2_	*S*_3_	*S*_4_	*S*_5_	*S*_6_	*S*_7_	Total
Polynomial kernel	79%	97%	96%	85%	94%	98%	99%	81%	91.51%
Sigmoid kernel	92%	94%	94%	69%	71%	96%	99%	91%	88.25%
Gaussian RBF kernel	81%	97%	95%	86%	92%	99%	100%	84%	91.76%
ML-kernel (*α* = 1)	81%	97%	95%	86%	92%	99%	100%	84%	91.76%
ML-kernel (0 < *α* ≤ 1)	92%	100%	96%	94%	98%	99%	100%	95%	96.75%
